# A Hybrid Vehicle Detection Method Based on Viola-Jones and HOG + SVM from UAV Images

**DOI:** 10.3390/s16081325

**Published:** 2016-08-19

**Authors:** Yongzheng Xu, Guizhen Yu, Yunpeng Wang, Xinkai Wu, Yalong Ma

**Affiliations:** 1 Beijing Key Laboratory for Cooperative Vehicle Infrastructure Systems and Safety Control, School of Transportation Science and Engineering, Beihang University, Beijing 100191, China; yongzhengxu@buaa.edu.cn (Y.X.); yugz@buaa.edu.cn (G.Y.); ypwang@buaa.edu.cn (Y.W.); mayalong@buaa.edu.cn (Y.M.); 2 Jiangsu Province Collaborative Innovation Center of Modern Urban Traffic Technologies, SiPaiLou #2, Nanjing 210096, China

**Keywords:** vehicle detection, unmanned aerial vehicle, Viola-Jones, HOG, road orientation

## Abstract

A new hybrid vehicle detection scheme which integrates the Viola-Jones (V-J) and linear SVM classifier with HOG feature (HOG + SVM) methods is proposed for vehicle detection from low-altitude unmanned aerial vehicle (UAV) images. As both V-J and HOG + SVM are sensitive to on-road vehicles’ in-plane rotation, the proposed scheme first adopts a roadway orientation adjustment method, which rotates each UAV image to align the roads with the horizontal direction so the original V-J or HOG + SVM method can be directly applied to achieve fast detection and high accuracy. To address the issue of descending detection speed for V-J and HOG + SVM, the proposed scheme further develops an adaptive switching strategy which sophistically integrates V-J and HOG + SVM methods based on their different descending trends of detection speed to improve detection efficiency. A comprehensive evaluation shows that the switching strategy, combined with the road orientation adjustment method, can significantly improve the efficiency and effectiveness of the vehicle detection from UAV images. The results also show that the proposed vehicle detection method is competitive compared with other existing vehicle detection methods. Furthermore, since the proposed vehicle detection method can be performed on videos captured from moving UAV platforms without the need of image registration or additional road database, it has great potentials of field applications. Future research will be focusing on expanding the current method for detecting other transportation modes such as buses, trucks, motors, bicycles, and pedestrians.

## 1. Introduction

Unmanned aerial vehicles (UAVs) have been widely used in many fields, such as chemical vapour detection [1], nature conservation monitoring [2] and wildlife emergency response [3]. Particularly, UAVs hold great promise for transportation applications, as demonstrated by many transportation studies [4,5,6,7]. One important application of UAV technology for transportation is to enhance the traffic and emergency monitoring which has been serving as a backbone of Intelligent Transportation Systems (ITS). Because UAVs are highly portable, UAVs can collect traffic data in the areas where the geographic locations of potential transportation-related problems are only crudely known, or conventional data collection technologies based on point detections cannot be applied to gather the data needed for transportation studies.

**Figure 1:**
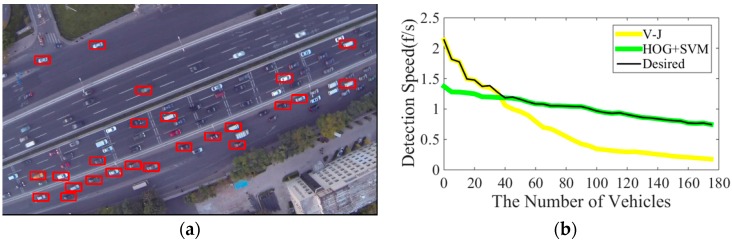
(**a**) V-J vehicle detection on an UAV image with unknown road orientation (red squares indicate successful detection); (**b**) Detection speeds for V-J and HOG + SVM (the black highlight line is the suggested hybrid method).

For traffic and emergency monitoring, one of the essential but challenging tasks is vehicle detection. Many traditional methods, like background subtraction [8], frame difference [9], and optical flow [10] have been applied for vehicle detections from UAV videos. However, these methods are sensitive to scene complexity and can only detect moving vehicles. Note fail detection of stopped vehicles has limited their applications for vehicle detection under congested traffic conditions. Also, these methods are sensitive to background motions. To improve vehicle detection, many new object detection algorithms have been proposed. Two most famous object detection schemes are the Viola-Jones (V-J) object detection scheme with Ada-boost classifier using Haar-like features [11] and linear support vector machine (SVM) using histogram of oriented gradients (HOG) features (HOG + SVM) [12]. A series of studies have demonstrated that these two methods achieved very promising results on vehicle detection [13,14,15,16,17]. However, when applying V-J and HOG + SVM methods to UAV images, the detection effectiveness and efficiency have been significantly downgraded due to the following two reasons:

(1) Both V-J and HOG + SVM are sensitive to objects’ in-plane rotation, therefore they only can detect vehicles when the orientations of vehicles are known and horizontal. Because vehicle orientations in UAV images are usually unknown and even changing, the detection accuracy (i.e., effectiveness) of these two methods has been significantly lowered. As shown in Figure 1a using original V-J method to detect vehicles in an UAV image with non-horizontal roadways, many vehicles cannot be detected. Note some methods have been proposed to address this issue (e.g., Jones & Viola [18], Cao et al. [14], Leitloff et al. [15], Moranduzzo and Melgani [19,20], Liu and Mattyus [21], etc.), but most of these methods are either time-consuming or need extra resources which limit their applications.
(2) The efficiency (i.e., detection speed) of both V-J and HOG + SVM is downgrading with the increase of the detection load (i.e., number of vehicles which need to be detected) in a frame. As shown in Figure 1b through our tests, the detection speeds of both methods are monotonically decreasing with the increase of the number of detected vehicles. But the descending rates of the detection speeds of these two methods demonstrate different characteristics. As shown in Figure 1b, the V-J method overall has a higher descending rate, but it detects much faster than HOG + SVM when the number of detected vehicles is relatively small. By contrast, HOG + SVM has lower detection speed than V-J when the number of detected vehicles is small, but it performs much better when the number of detected vehicles is large. These different characteristics suggest an intuitive idea that the overall efficiency could be improved by switching these two methods based on the number of vehicles which need to be detected, as the black line suggested in Figure 1b.

This research aims to improve the effectiveness and efficiency of vehicle detection from UAV images by addressing the above mentioned two issues. Particularly, this research proposes a new hybrid vehicle detection scheme which integrates both V-J and HOG + SVM methods. The proposed scheme has two unique features which are designated to solve the above mentioned two issues:

(1) To address the challenge that both V-J and HOG + SVM are sensitive to on-road vehicles’ in-plane rotation, we adopt a roadway orientation adjustment method. The basic idea of this method is to first measure the orientation of the road, and then rotates the road according to the detected orientation so the road and on-road vehicles will be horizontal after rotation so the original V-J or HOG + SVM methods can be applied to achieve fast detection and high accuracy. More importantly, different with some existing solutions for the issue of unknown road orientation (e.g., [14,15,18]), the proposed road orientation adjustment method does not need any additional extra resource and only needs to rotate the image one time, so the new method significantly saves computational time and reduces false detection rates.
(2) To address the issue of descending detection speeds for both V-J and HOG + SVM and achieve better efficiency, we integrate V-J and HOG + SVM methods based on their different descending trends of detection speed and propose a hybrid and adaptive switching strategy which sophistically searches for, if not the optimal, at lease improved solution, by switching V-J and HOG + SVM detection methods based on the change of detection speed of these two methods during the detection. This switching strategy, combined with the road orientation adjustment method, significantly improves the efficiency and effectiveness of vehicle detections from UAV images.

The rest of the paper is organized as follows: Section 2 briefly reviews some background information by introducing the basic concepts of V-J and HOG + SVM methods, followed by the methodological details of the proposed hybrid vehicle detection scheme in Section 3. Section 4 presents a comprehensive evaluation of the proposed method using diverse scenarios. Section 5 presents a discussion on some extensions and limitations of the proposed method. Finally, Section 6 concludes this paper with some remarks.

## 2. Background

A large amount of research has been performed on vehicle detection from UAV images over the years. Many of them applied some traditional methods, such as background subtraction, frame difference, optical flow, etc. For example, Azevedo [8] applied a median-based background subtraction method to fast detect vehicles; Shastry and Schowengerdt [9] applied a frame difference method, combining with the image registration process to detect moving vehicles; and Yalcin [10] proposed a motion-based optical flow method to detect moving vehicles. However, methods like frame difference, background subtraction and optical flow are sensitive to scene complexity therefore have difficulties in detecting slow-moving or stopped vehicles when traffic is congested. Also, some methods, like optical flow method, are sensitive to background motions.

In recent years, object detection algorithms have become popular for vehicle detection from UAV videos. Generally speaking, object detection algorithms are less sensitive to image noise, background motions and scene complexity, therefore are more robust for vehicle detections from UAV videos. For example, Viola and Jones [11] developed the famous V-J object detection scheme; Cao et al. [13] applied the SVM using HOG features for vehicle detection; Leitloff et al. [15] proposed a V-J-based two-stage method to improve detection results; Tuermer et al. [16] used Disparity Maps to limit the search space to road regions and then applied a standard HOG detector to detect vehicles; and Felzenszwalb [22] developed an objects detection framework by applying discriminatively trained deformable part model (DPM). Among them, two most widely applied methods are the V-J and HOG + SVM methods. However, these two methods have two major issues which limit their applications and downgrade their performance as mentioned above. This research aims to improve these two methods. Before we discuss the details of our method, we will briefly overview the background theories of these two methods first.

### 2.1. Viola-Jones Object Detection Scheme

**Figure 2:**
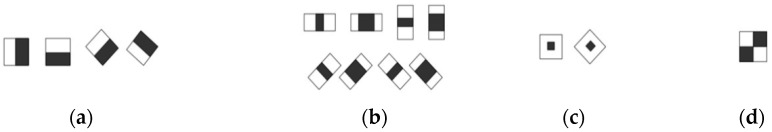
Haar-like features. (**a**) Edge features; (**b**) Line features; (**c**) Center-surround features; (**d**) Special diagonal line features.

The V-J scheme is based on multiple cascaded Haar-like classifiers [11]. The basic concept is to use a conjunctive set of weak classifiers to form a strong classifier. The core of this scheme is the Haar-like features, which are essentially drawn from the spatial response of Haar basis functions and derivatives to a given type of feature at a given orientation within the image. In practice, Haar-like features are computed as the sum of differences of the pixel intensities between different rectangular regions at a specific location in a detection window (Figure 2). Rectangular features can be computed very rapidly using an intermediate representation of the image called integral image (also called summed area table [11]). However, these individual Haar-like features are weak discriminative classifiers, which only give the right answer a little more often than a random decision. To construct a “strong” discriminative classifier, many “weak” classifiers are combined as a conjunctive cascade; and Gentle AdaBoost [23], a machine learning meta-algorithm, is applied to train a cascaded classifier over a set of thousands of positive and negative training images.

The evaluation of the strong classifiers generated by the AdaBoost learning process can be done quickly, but it is not fast enough to process in real-time. For this reason, the strong classifiers are arranged in a cascade in order of complexity. In each cascade, each successive classifier is trained only on those selected samples which pass through the preceding classifiers. If at any stage in the cascade a classifier rejects the sub-window under inspection, no further processing is performed. The cascade therefore has the form of a degenerate tree. This degenerative decision-tree structure can eliminate negative regions as early as possible during detection to focus attention on promising regions of the image. Therefore, this detection strategy dramatically increases the processing speed of the detector, provides an underlying robustness to changes in scale, and maintains achievable real-time performance.

**Figure 3:**
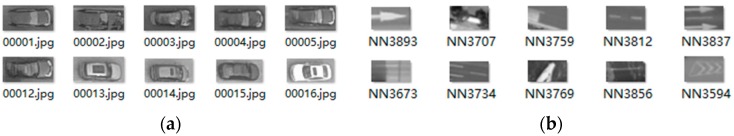
Samples images. (**a**) Positive training samples; (**b**) Negative training samples.

Before applying the V-J method, the critical first step is to build a sample library which provides a training set to include both positive and negative images. As mentioned in [24,25], the number of samples plays a key role in training classifier. In our paper, over 16,800 positive samples (see Figure 3a) were manually collected from 600 UAV images extracted from 100 min of the video data (one image per 10 s). From each UAV image, about 10–100 vehicles under different traffic conditions were manually extracted to form 16,800 positive training samples. These 16,800 samples do not contain duplicated ones and each sample only contains one vehicle. Note that some samples which contain the same vehicle but different backgrounds are treated as different positive samples. All vehicle samples were rotated to align with the horizontal direction. In addition, over 26,000 negative samples (see Figure 3b) which do not contain vehicles were also manually collected.

All positive images were further transformed into gray scale images and normalized to a compressed size of 40 × 20 during our tests. Each image was used to calculate a complete set of Haar-like features, and in total, 479,430 Haar-like features were extracted from all images. These 479,430 features were further trained into 469 most significant features by applying the Gentle AdaBoost algorithm [23]. Finally, an 18-stage cascaded classifier was formed based on the 469 significant Haar-like features.

### 2.2. Linear SVM Classifier with HOG Feature

The histogram of oriented gradients (HOG) is a feature descriptor used in computer vision and image processing for the purpose of object detection. HOG was first described by Dalal and Triggs [12] and achieved good performance for pedestrian detection. The HOG descriptor has many advantages, for example, it is invariant to geometric and photometric transformations, since it operates on local cells. Generally, the extraction of HOG features includes five steps [26] as described as follows:

Step 1: Gradient computation. Step 1 computes the gradient values and orientations of all pixel units in the image by applying the 1-D centered point discrete derivative mask with the filter kernel [−1, 0, 1] in one or both of the horizontal and vertical directions.
Step 2: Orientation binning. Step 2 is to create the cell histograms. In this step, the image was divided into cells, and the 1-D histogram $H_{i}$ is generated by binning local gradients according to the orientation of each cell. Each pixel within the cell will cast a weighted vote for an orientation-based histogram channel based on the values found in the gradient computation.
Step 3: Descriptor blocks. Step 3 groups cells together into larger, spatially connected blocks $F_{i}$.
Step 4: Block normalization. Step 4 is to normalize blocks in order to account for changes in illumination and contrast. A cell can be involved in several block normalizations for the overlapping block, since each block consists of a group of cells. By concatenating the histograms of all blocks, the feature vector $V_{HOG}$ is obtained. The HOG descriptor is then the concatenated vector of the components of the normalized cell histograms from all the block regions.
Step 5: SVM classifier. The final step is to feed the descriptors into the SVM classifier. SVM is a binary classifier which looks for an optimal hyperplane as a decision function.

**Figure 4:**
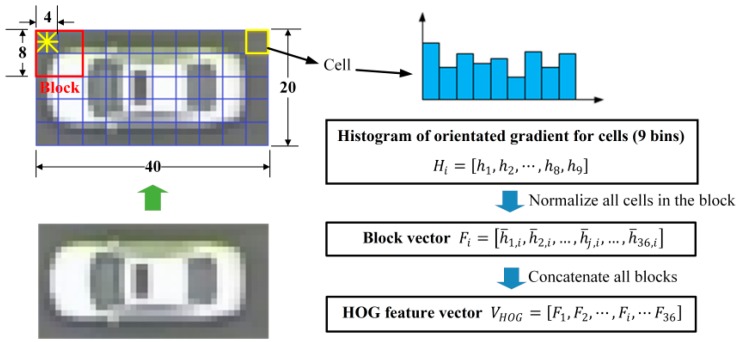
The flowchart to obtain HOG descriptor.

HOG + SVM will use the same sample library (16,800 positive samples and 26,000 negative samples) as for the V-J vehicle detector. To extract the HOG feature, each sample image needs to be first normalized to a compressed size of 40 × 20 pixels; then blocks (8 × 8) with 4-pixel stride step and 9 histogram bins (each bin is corresponding to 20° of orientation) were used to calculate the HOG feature vector. So for an image with 40 × 20 pixels, a total of 36 blocks can be identified (note 36 is calculated by $\left(\frac{\left(40\;-\;8\right)}{4}+1\right)\times \left(\frac{\left(20\;-\;8\right)}{4}+1\right)=36$). The final HOG feature descriptor can be described by:

(1) $$V_{HOG}=\left[F_{1},F_{2},\cdots ,F_{i},\cdots F_{36}\right]$$

where $V_{HOG}$ is the HOG descriptor, and $F_{i}$ is the normalized block vector for i-th block. As each block contains four cells, and each cell contains nine bins, $F_{i}=\left[{\bar{h}}_{1,i},{\bar{h}}_{2,i},\ldots ,{\bar{h}}_{j,i},\ldots ,{\bar{h}}_{36,i}\right]$, where ${\bar{h}}_{j,i}$ is the j-th normalized value in i-th block. Figure 4 shows the flowchart to obtain HOG descriptor.

### 2.3. Limitations

V-J and HOG + SVM have been widely applied in many fields, but one of critical issues of these methods is that both methods are sensitive to object’s in-plane rotation. So when applying them to vehicle detection, especially for UAV images, many vehicles with unknown orientations cannot be detected. A simple way to address this issue is to rotate images. For example, Cao et al. [14] rotated each video image nine times (each time 20 degree) in order to cover 180 degrees, but repeating detections of the same image significantly increase the detection time and lead to more false detections. Some researchers tried to train multiple detectors for objects of different angles. For example, Viola and Jones [18] built 12 different detectors for face detection to cover different views. However, training multiple detectors brings heavy workload. Leitloff et al. [15], on the other hand, used road database to get the road orientation and rotated the image according to the road orientation. Because on-road vehicles run in the same direction with the road, the roads and on-road vehicles in rotated images will be aligned with the original vehicle detector; therefore, the original V-J or HOG + SVM can be applied. However, the need of additional geometric information limits its applications. Another issue of V-J and HOG + SVM is the descending detection speed with the increase number of detected vehicles. As shown in Figure 1b, the detection speeds of both methods are monotonically decreasing as the number of detected vehicles increases. However, the descending rates of the detection speeds of these two methods demonstrate different characteristics. Particularly, V-J method detects much faster than HOG + SVM when the number of detected vehicles is relatively small; by contrast, HOG + SVM performs better when the number of detected vehicles is large. The proposed method will take advantage of these features to develop a hybrid vehicle detection scheme which integrates V-J and HOG + SVM to achieve better efficiency and effectiveness.

## 3. Methodology

### 3.1. Overall Framework

This research aims to address two critical issues of V-J and HOG + SVM by developing a hybrid vehicle detection scheme. In detail, the proposed scheme integrates the following two improvements:

(1) A roadway orientation adjustment method. The proposed detection scheme adopts a roadway orientation adjustment method to address the roadway rotation issue. The idea is straightforward: first, measure the orientation of the road using the line segment detector (LSD) [27]; second, rotate the road according to the detected orientation so the road and on-road vehicles will be horizontal after rotation; and third, apply the original V-J or HOG + SVM methods to achieve fast detection and high accuracy. A highlight of this approach is that the proposed road orientation adjustment method only needs to rotate the image one time and does not need any additional extra resource. Therefore, the new method significantly saves computational time and reduces false detection rates.
(2) A detector switching strategy. The proposed scheme further develops an adaptive switching strategy to integrate V-J and HOG + SVM methods based on their different descending trends of detection speed in order to improve the efficiency. Basically, this strategy “intelligently” switches the detection methods between V-J and HOG + SVM to choose the one which has faster detection speed. As shown in Figure 1b, the detection speed essentially is determined by the workload, i.e., the number of vehicles which need to be detected; and V-J and HOG + SVM shows different characteristics of detection speed. So the proposed switching method will detect the detection speeds of both methods periodically and choose the one with faster detection speed.

**Figure 5:**
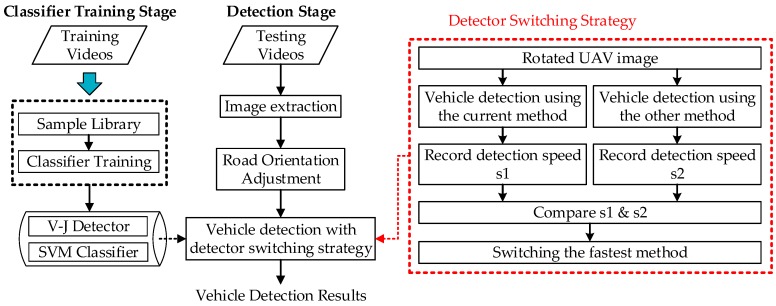
Proposed vehicle detection framework.

The overall framework of the proposed vehicle detection method is shown in Figure 5. The details of the above mentioned two major functions will be introduced in the following sections.

### 3.2. Road Orientation Adjustment Method

As mentioned before, when directly applying V-J or HOG + SVM to detect vehicles from UAV images, the detection rate is significantly low. The reason, as mentioned before, is that the original V-J and HOG + SVM schemes can only detect vehicles with orientations which are aligned with the detectors (i.e., vehicles in training sets). To address this issue, this research proposes a road orientation adjustment method.

**Figure 6:**
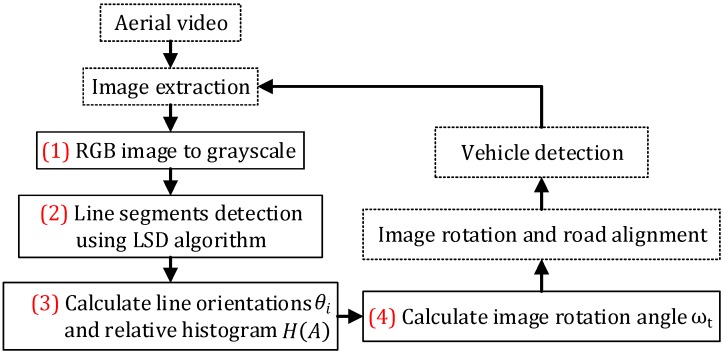
Flowchart of the proposed road orientation adjustment method.

**Figure 7:**
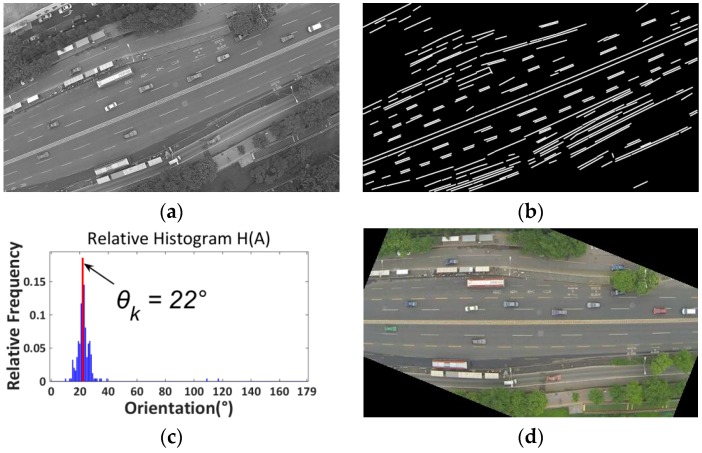
(**a**) Grayscale image; (**b**) Line segments detection; (**c**) Relative histogram (blue bins: distribution frequencies of relative histogram which correspond to different angles; red bin: the maximum distribution frequency of relative histogram which corresponds to the orientation of the road); (**d**) Rotated image.

Essentially, the proposed road orientation adjustment method is to rotate the image according to the orientation of the road, i.e., the angle between the road and the horizontal of the image. After rotation, the road and on-road vehicles will be aligned with the vehicle detectors. As shown in Figure 6, the general procedure includes:

(1) First, original color images are extracted from aerial videos and then transformed into gray scale images (Figure 7a);
(2) Second, the LSD algorithm [27] is applied to detect straight edge segments (Figure 7b);
(3) Third, the orientation of each detected line segment $\theta_{i}$ is calculated and the relative histogram H(A) of these line orientations is derived (Figure 7c). The angle corresponding to the maximum distribution frequency of relative histogram will be considered as the orientation of the road;
(4) Last, to minimize in-plane rotation jitters, the final rotation angle $\omega_{t}$ for frame t is smoothed by the first-order lag filtering algorithm, which considers the rotation angle $\omega_{t-1}$ for the last frame t−1. After rotation, the directions of roads and on-road vehicles are aligned with the horizontal direction of the image (Figure 7d). The details of some key techniques will be elaborated in the following.

**Figure 8:**
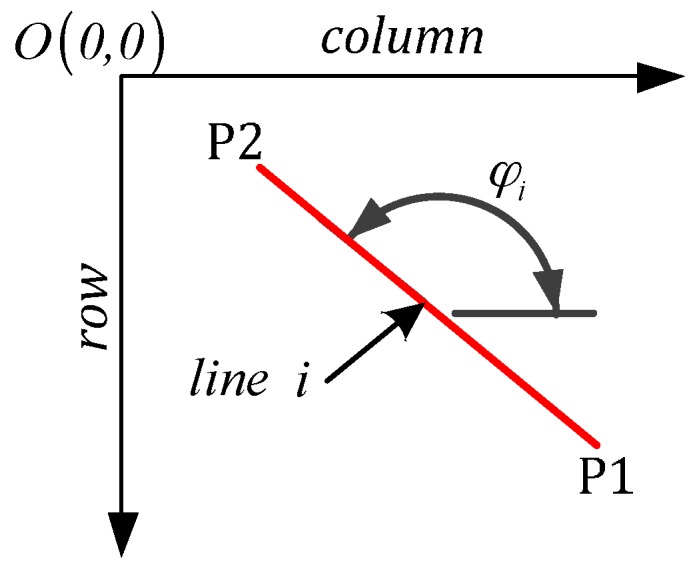
Orientation $\varphi_{i}$ of line i.

*(1) Straight Line Segments Detection*: The proposed method first applies the LSD algorithm [27] to detect straight line segments. LSD is a linear-time line segment detector giving subpixel accurate results. Unlike other line detection algorithms, such as Hough transform [28], which requires tedious parameter tunings and is very likely to be affected by other redundant edges, the LSD algorithm can work on digital image without parameter tuning, therefore it is more robust and efficient. The LSD algorithm is open source [29] and the implementation of this algorithm is available in the Open Source Computer Vision (OpenCV) version 3.1 (Nizhny Novgorod, Russia). As shown in Figure 7b, many line segments are detected. The orientation of each detected line segment can be estimated as described in Equation (2):

(2) $$\varphi_{i}=\{\begin{array}{cc} arctan\left(-\frac{r_{i\_2}-r_{i\_1}}{c_{i\_2}-c_{i\_1}}\right), & c_{i\_1}\neq c_{i\_2}\\ 90{}^{\circ}, & c_{i\_1}=c_{i\_2} \end{array}$$

where $\varphi_{i}$ is the orientation of detected line i; $\varphi_{i}$ is an integer and $\varphi_{i}\in \left[0{}^{\circ},180{}^{\circ}\right)$; $\left(c_{i\_1},r_{i\_1}\right)$ and $\left(c_{i\_2},r_{i\_2}\right)$ represent the pixel coordinates of the start and end points (P1 and P2, see Figure 8) of line i in the image coordinate system.

*(2) Road Orientation Estimation by Relative Histogram*: As shown in Figure 7b, the road is parallel to the majority of detected line segments. Therefore, to estimate the orientation of the road, essentially, we need to identify the angle of the majority of line segments which have similar orientations. More precisely, we aim to identify an angle range of 1°. A relative frequency histogram is applied to identify the angle. The detailed steps are described as the following:

Step 1: Identify n: the total number of lines;
Step 2: Define 180 class intervals: $\theta_{1}=\left[0{}^{\circ},1{}^{\circ}\right),\;\theta_{2}=\left[1{}^{\circ},2{}^{\circ}\right),\ldots ,\;\theta_{i}=\left[\left(i-1\right){}^{\circ},i{}^{\circ}\right),\ldots ,\theta_{180}=\left[179{}^{\circ},180{}^{\circ}\right)$;
Step 3: Determine the frequency,$\;h\left(\theta_{i}\right)$, i.e., the number of lines with the angle within the angle interval of class $\theta_{i}$;
Step 4: Calculate the relative frequency (i.e., proportion) of each class by dividing the class frequency by the total number n in the sample, i.e., $H\left(\theta_{i}\right)=h\left(\theta_{i}\right)/n$;
Step 5: Draw a rectangle for each class with the class interval as the base and the height equal to the relative frequency of the class to form a relative histogram (Figure 7c);
Step 6: Identify $\theta_{k}$, which is corresponding to the highest rectangle in relative histogram, and Θ=k° is considered as the orientation of the road.

*(3) Image Rotation Angle Estimation by First-Order Lag Filtering*: To minimize the impact of the jitters caused by UAVs, the first-order lag filtering algorithm is further applied to calculate the weighted average of the estimated road orientations of current and previous frames. The final image rotation angle for current frame j, is calculated by:

(3) $$\omega_{j}=\left(1-w\right)\times \omega_{j-1}+w\times \Theta$$

where $\omega_{j-1}$ is the image rotation angle for the last frame j−1, and w is a predetermined weight.

**Figure 9:**
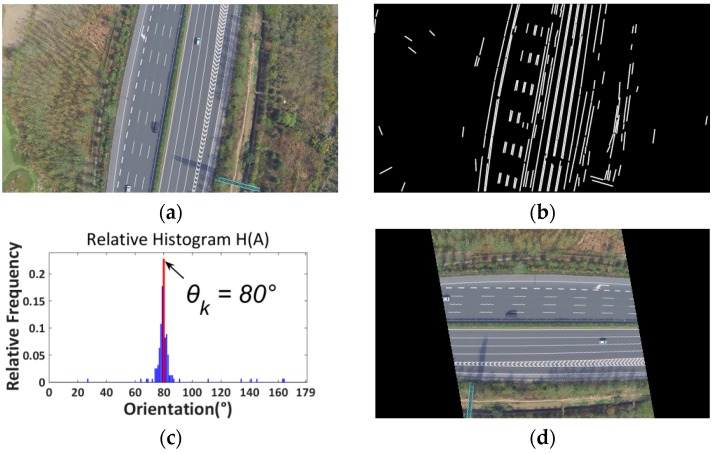
Suburban road. (**a**) Color image; (**b**) Line segments detection using LSD; (**c**) Relative histogram (blue bins: distribution frequencies of relative histogram which correspond to different angles; red bin: the maximum distribution frequency of relative histogram which corresponds to the orientation of the road); (**d**) Rotated image.

The final step is to rotate the image by $\omega_{j}$. After rotation, the road will become horizontal. Figure 9 presents another example for a suburban road.

**Figure 10:**
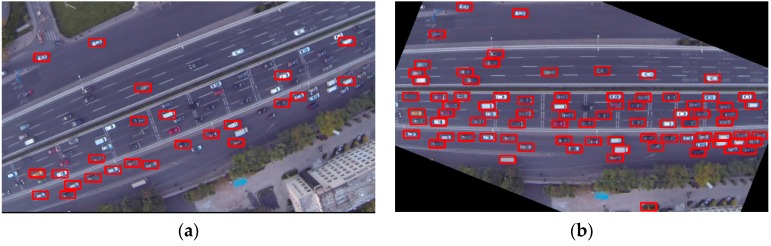
Vehicle detections using the V-J vehicle detector: (**a**) without road orientation adjustment; (**b**) with road orientation adjustment.

A highlight of this method is that each UAV image only needs to be rotated once. A visual comparison of vehicle detections using V-J scheme without and with road orientation adjustment is presented in Figure 10. Detected vehicles are marked with red rectangles. As shown in Figure 10a, because the orientation of the road is not horizontal, many vehicles could not be detected by the V-J method. On the contrary, as shown in Figure 10b, after applying the pre-processing step of road orientation adjustment, most vehicles can be detected. Detailed evaluation will be presented in Section 4.

### 3.3. Detector Switching Strategy

**Figure 11:**
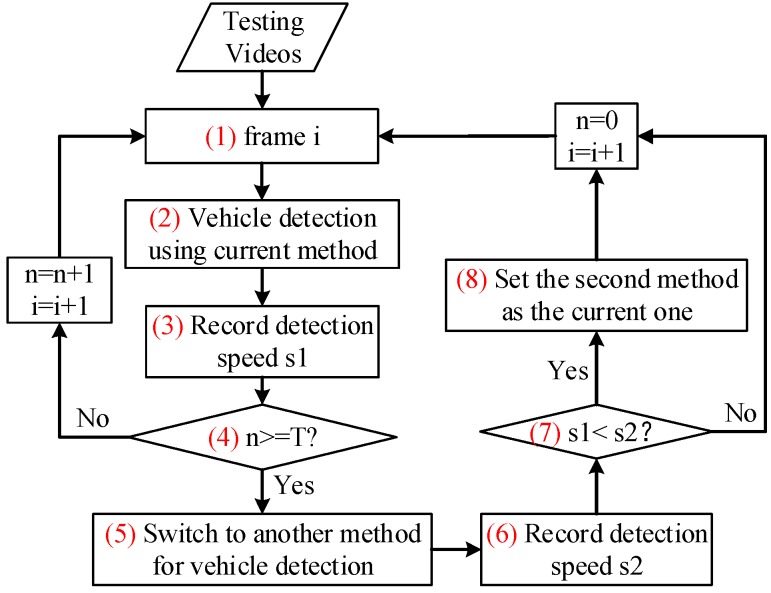
Flowchart of the proposed detector switching strategy.

The proposed vehicle detection scheme further adopts a vehicle detector switching strategy to improve detection speed. This strategy is based on the speed characteristic lines (see Figure 1b) of the V-J and HOG + SVM methods. The comparison between the detection speeds of both methods (Figure 1b) shows that when the number of vehicles in an UAV image is small, V-J should be applied to achieve faster detection speed, while when the number of vehicles in an UAV image is large, HOG + SVM should be selected to gain better detection speed. Based on this observation, we propose a switching strategy which can “intelligently” choose the faster detection method between V-J and HOG + SVM during the detection. The idea is straightforward. Since we won’t be able to know the number of vehicles in the image until we finish detecting, an intuitive idea is to directly compare the detection speeds of both methods and choose the one with faster detection speed. But performing both methods to each frame could be time consuming and is really not necessary. Also, since traffic conditions (i.e., the number of vehicles in the image) are relatively stable within a short period (such as a few seconds) based on the research in [30], it would be much more reasonable and efficient to switch detection methods every several seconds (note there are 24 frames each second). Figure 11 presents the overall flowchart of the proposed switching strategy.

From the flowchart, we can see that for any frame i, the proposed switching strategy first detects vehicles in the image using the current detection method, i.e., the detection method used to detect vehicles for previous frame i−1 (i.e., step (2) in the flowchart). After detection, the detection speed, s1, will be recorded (i.e., step (3) in the flowchart). Then the program will check if the accumulative number of frames (n) which apply the current detection method for vehicle detection has reached a predetermined cycle value T (i.e., step (4) in the flowchart). If NO, then the program moves to frame i+1 and repeat steps (2)–(4); and if YES, the program will apply the other detection method to detect the vehicles in frame i again (i.e., step (5) in the flowchart) and record the detection speed as s2 (i.e., step (6) in the flowchart). If s1<s2 (i.e., step (7) in the flowchart), the program will switch to the new detection method and set it as the “current” method (i.e., step (8) in the flowchart) and apply it to detect vehicles for the following T frames. Otherwise, if s1≥s2, the program will keep applying the current method to detect vehicles for the following T frames, i.e., repeat previous steps. To be clear, only the image of the $T_{th}$ frame needs to be detected twice, other T−1 images from previous T−1 frames need to be detected only once. Section 4 presents the testing results. Note during our testing, the value of T is set as 24 (namely, the detection speed comparison is conducted every 1 s). Furthermore, a sensitivity analysis has been conducted to evaluate the impact of different values of T in Section 5.

## 4. Evaluation

### 4.1. UAV Data Collection

**Figure 12:**
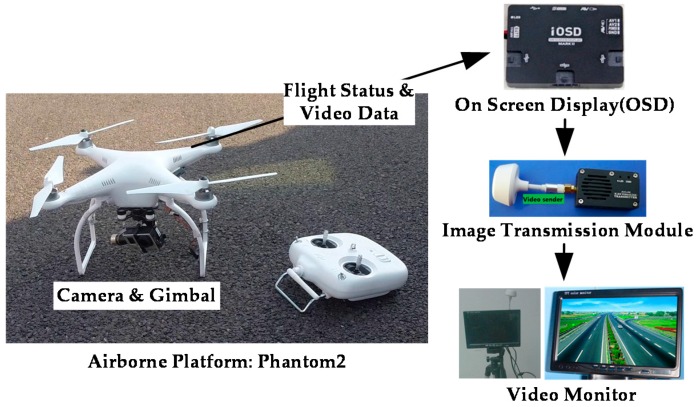
UAV system architecture.

The UAV system used in this research is equipped with a quadcopter (model: Phantom 2) airborne platform and a Gopro Hero Black Edition 3 aerial camera (see Figure 12). A 3-axis gimbal is mounted on the UAV to stabilize the videos and eliminate video jitters caused by UAV therefore greatly reducing the impact from external factors, such as wind. In addition, an On-Screen Display (OSD), an image transmission module and a video monitor are installed in the system for data transmission and airborne flying status monitoring and control.

**Figure 13:**
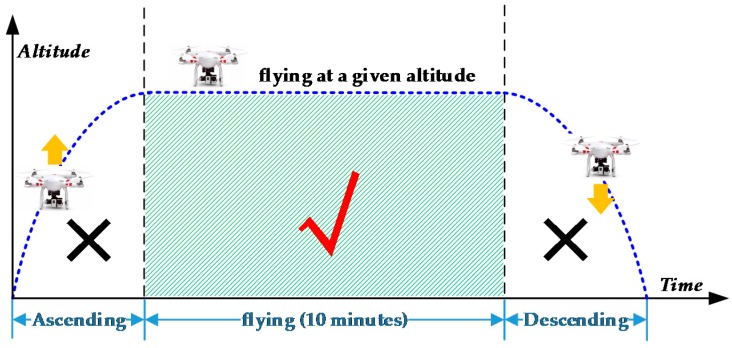
Altitude-Time graph of the quadcopter.

The evaluation was based on low-altitude UAV videos captured from five different scenarios with diverse traffic and weather conditions (Table 1). These diverse testing scenes are specifically chosen in order to test the effectiveness of the proposed method. For each scenario, three 15-min videos were recorded, but only 10-min video in the middle were used due to the UAV ascent and descent (Figure 13), so the total video time for each scenario is 30 min. Among them, 20 min of videos were chosen for building the sample library; and the remaining 10 min were used for testing. The resolution of the videos is 1920 × 1080 and the frame rate is 24 frames per second (f/s). Note, all UAV videos were captured with the UAV hovering over a fixed location. Due to UAV motions, the orientations of the roads and on-road vehicles in the images are unknown and changing frequently.

During evaluation, in order to avoid the situation where the same vehicle has been detected multiple times in different frames, we extract detection images each 20 s from the 10-min video for comparison. Because the length of the road segment in an image is about 160 meters, most likely a vehicle will pass the road segment in 20 s. This significantly reduces the possibility of one vehicle being detected multiple times. Note when traffic is congested, it is still possible that some slow-moving vehicles will be detected more than once. All the experiments were conducted using C++ implementation on a laptop computer (model: ThinkPad T440P, Lenovo, Beijing, China) with an Intel i5-4300M @ 2.60 GHz CPU and 8 GB DDR3 memory.

### 4.2. Performance Evaluation

The performance of vehicle detection is evaluated by the following four typical indicators: *detection speed* (f/s), *Correctness*, *Completeness*, and *Quality*, defined in Equation (4):

(4) $$\begin{array}{l} Correctness(Cor.)=\frac{true\;positives}{true\;positives+false\;positives}\\ Completeness(Com.)=\frac{true\;positives}{true\;positives+false\;negatives}\\ Quality(Qua.)=\frac{true\;positives}{true\;positives+false\;positives+false\;negatives} \end{array}$$

where *true positives* are the number of “true” detected vehicles; *false positives* are the number of “false” detected objects which are non-vehicle objects; *false negatives* are the number of vehicles missed. In particular, *Quality* is considered as the strictest criterion, which contains both possible detection errors (false positives and false negatives). Note that a successful “detection” is defined as a correct detection of a vehicle in one frame.

### 4.3. Results and Comparison

Conceptually, by incorporating the road orientation adjustment method, the proposed vehicle detection method will be insensitive to road orientation changes and therefore can achieve high *Completeness* and *Quality*. Furthermore, by combining the vehicle detector switching strategy, the proposed method can achieve fast vehicle detection. To fairly verify these two points, the proposed method is compared with nine other methods:

(1) ViBe, a universal background subtraction algorithm [31];
(2) Frame difference [9] (referred as Frame Diff in Table 2);
(3) Original V-J method (referred as V-J in Table 2) [11];
(4) Rotate each image every 20° from 0° to 180° and detect nine times using the original V-J method (referred as V-J + 9 in Table 2) [14];
(5) Original V-J method combines with the proposed road orientation adjustment method only (referred as V-J + R in Table 2);
(6) Original HOG + SVM (referred as SVM in Table 2) [12];
(7) Rotate each image every 20° from 0° to 180° and detect nine times using the original HOG + SVM method (referred as SVM + 9 in Table 2) [14];
(8) Original HOG + SVM method combines with the proposed road orientation adjustment method only (referred as SVM+R in Table 2);
(9) Apply the proposed vehicle detector switching strategy to integrate V-J and HOG + SVM (without road orientation adjustment) (referred as V-J + SVM + S in Table 2);
(10) Apply the proposed vehicle detector switching strategy and road orientation adjustment method to integrate V-J and HOG + SVM (the proposed method, V-J + SVM + R + S).

As ViBe [31] and frame difference [9] are sensitive to background motions, image registration [9] is performed first to compensate UAV motions. This registration process converts the spatio-temporal video into temporal information, thereby can correct UAV motion and attitude errors. The time for image registration is included in the detection time for these two methods.

Also, as mentioned above, for each scenario, a 10-min video with the resolution of 1920 × 1080 was used for testing. The detection speed for each method was computed as an average of each 10-min video. Note vehicle detection was performed on the entire image (1920 × 1080). After detection, we only extracted images every 20 s in order to avoid that the same vehicle in different frames has been detected multiple times. So for each scenario, totally of 30 detected images were used for computing *Correctness*, *Completeness*, and *Quality*.

The testing results of ten methods are presented in Table 2. The average metrics listed in the bottom of Table 2 show that our method achieved the best *Quality* (82.32%) with fast speed (1.17 f/s). Some comparisons are presented as follows:

(1) *Vibe & Frame Difference*: These two methods achieved fast detection speed but with low *Quality* (54.24% & 49.03%) which are too low to be accepted for real-world applications. The reason is that some non-vehicle objects (such as tricycles and moving pedestrians) lead to many false positives. Besides, slow-moving or stopped vehicles and some black vehicles which have similar colors with the road surface cannot be detected during detection.
(2) *V-J vs. V-J + 9*: The *Completeness* (76.91%) of V-J is low, this is because many vehicles that are not parallel to horizontal cannot be detected, thus generating many false negatives. The *Completeness* (92.31%) of V-J + 9 is significantly higher than the original V-J. After images were rotated every 20° from 0° to 180° and detected 9 times, vehicles of different orientations can be detected. However, repeating detections of the same image lead to more false positives and greatly increase detection time. The *Correctness* of V-J + 9 (76.10%) is lower than that of the original V-J (83.22%). The *detection speed* of V-J + 9 (0.079 f/s) is also significantly slower than V-J (1.14 f/s).
(3) *SVM vs. SVM + 9*: The comparison of SVM and SVM + 9 also demonstrates similar results as V-J vs. V-J + 9. SVM + 9 achieved higher *Completeness* (89.21%) than SVM (73.01%) but with low *Correctness* and *detection speed*.
(4) *V-J vs. V-J + R*: V-J + R achieves higher *Completeness* (92.37%) than V-J (76.91%); because by incorporating road orientation adjustment, on-road vehicles of unknown orientations will be aligned with the horizontal direction which can be detected by the original V-J detector. V-J + R achieves higher *Quality* (82.09%) than V-J (66.79%), but the detection speed of V-J + R is slower than the original V-J due to two reasons: (1) the road orientation adjustment step will cost some time for road orientation detection and image rotation; and (2) after image rotation and road alignment, many more vehicles in the UAV image need to be detected.
(5) *SVM vs. SVM + R*: The method SVM + R also achieves higher *Quality* (81.53%) than the original SVM method (64.28%). Similar to V-J + R, the detection speed of SVM + R is slower than the original SVM.
(6) *V-J + R vs. V-J + 9*: V-J + R achieves slightly higher *Completeness* (92.37%) than V-J + 9 (92.31%), because those rotated vehicles in V-J + 9 are in fact not exactly aligned with the horizontal direction, therefore may not adapt the original V-J detector well. Also, V-J + R achieves faster detection speed (0.88 f/s) than V-J + 9 (0.079 f/s). The comparisons demonstrate that the proposed road orientation adjustment method can improve both the *Completeness* and *Quality* compared with the original V-J and HOG + SVM methods, but leads to a slightly slower detection speed.
(7) *SVM + R vs. SVM + 9*: Similarly, SVM + R achieved higher *Completeness* (91.13%) than SVM + 9 (89.21%) and higher detection speed.
(8) *V-J vs. SVM vs. V-J + SVM + S*: The proposed switching method (i.e., V-J + SVM + S) achieves faster detection speed (1.27 f/s) than both the original V-J (1.14 f/s) and SVM (1.07 f/s) methods, because the proposed switching strategy can automatically choose the faster method between V-J and HOG + SVM during the detection. Note that, without the road orientation adjustment, the proposed switching method only achieves low *Quality* (65.08%), which is similar to V-J (66.79%) and SVM (64.28%).
(9) *V-J + SVM + R + S*: Our method, which combines the road orientation adjustment method, achieves the best *Quality* (82.32%) than other nine methods. The detection speed of our method is slower than V-J + SVM + S, which is a trade-off between high *Quality* and fast *detection speed*. However, our method is still faster than the original V-J and SVM methods. The detection speed of 1.17 f/s is acceptable for real-time applications.

Overall, the proposed method achieves good vehicle detection performance with fast speed. Particularly, our method is insensitive to on-road vehicles’ in-plane rotation. Therefore, the proposed method can be performed on videos captured from moving UAV platforms (for example, UAVs flying along the road) without the need of image registration [9,31] or additional road database [15,16], thus has great potentials in wild field applications.

## 5. Discussion

### 5.1. Road Orientation Adjustment Method for Roadways with More Than One Orientation

**Figure 14:**
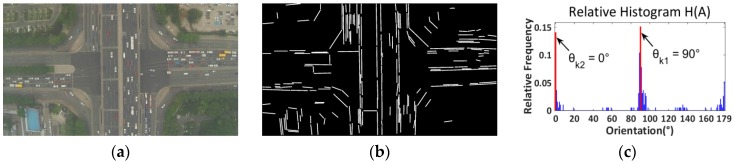
Roads orientation detection for an interchange: (**a**) Color image; (**b**) Line segments detection using LSD; (**c**) Relative frequency histogram (blue bins: distribution frequencies of relative histogram which correspond to different angles; red bins: two maximum distribution frequencies of relative histogram which correspond to two orientations of an interchange).

**Figure 15:**
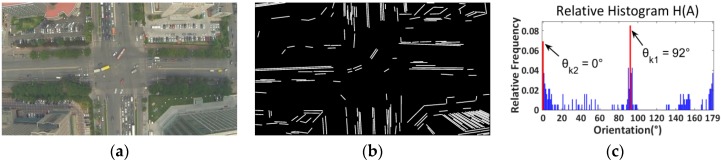
Roads orientation detection for an intersection: (**a**) Color image; (**b**) Line segments detection using LSD; (**c**) Relative frequency histogram (blue bins: distribution frequencies of relative histogram which correspond to different angles; red bins: the first two maximum distribution frequencies of relative histogram which correspond to two orientations of an intersection).

The proposed road orientation adjustment method can be applied for roadways with more than one orientation. Here we present some testing results for roads with two orientations. As shown in Figure 14 and Figure 15, the proposed road orientation adjustment method was applied to detect road orientations for: (1) an interchange with one freeway crossing over an arterial; and (2) a regular 4-leg intersection. As shown in the figures, two peaks were found in the relative frequency histograms. These two peaks essentially indicate the orientations of roads. For the case of interchange (Figure 14), two different orientations are around 0° and 90°; and for the case of 4-leg intersection (Figure 15), the orientations for the two orientations are around 0° and 92°. Note conceptually the method can be applied to detect many orientations. But when the road orientations are more than two, the peaks in the relative histogram could be difficult to identify.

Besides, it should be mentioned that the proposed road orientation adjustment method can only extract road orientations of straight roads. It will be difficult to extract the orientations of curve roads or very smaller roads. This also leads to some false detections (false negatives and false positives) during our vehicle detection when applying our method to detect vehicles on curve or small roads.

### 5.2. Straight Line Detection Using Other Algorithms

**Figure 16:**
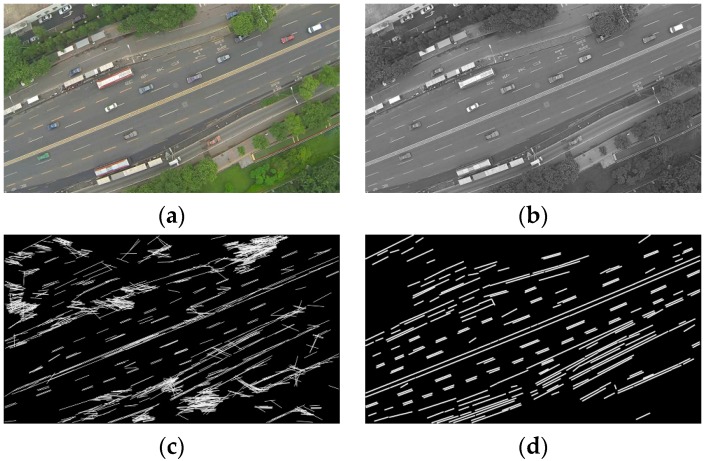
Line segments detection. (**a**) Color image; (**b**) Grayscale image; (**c**) Canny followed by Hough transform; (**d**) line segment detector (LSD).

In this paper, we also compared the adopted LSD method with other line detection algorithms [28,32,33]. Particularly, we compared the LSD method to the Canny edge detector [34] followed by a Hough transform [28]. Note the Hough transform method need to tune parameters for each image manually, because using fixed parameters can lead to many false positives or false negatives. The comparison result is presented in Figure 16. As shown in the figure, the adopted line segment detector (LSD) can achieve much better performance. As seen in Figure 16c, many “redundant” lines were detected by Hough transform; by contrast, LSD achieved very “clean” line detection results (Figure 16d).

### 5.3. Road Orientation Adjustment on Imagery with Low Radiometric Quality

**Figure 17:**
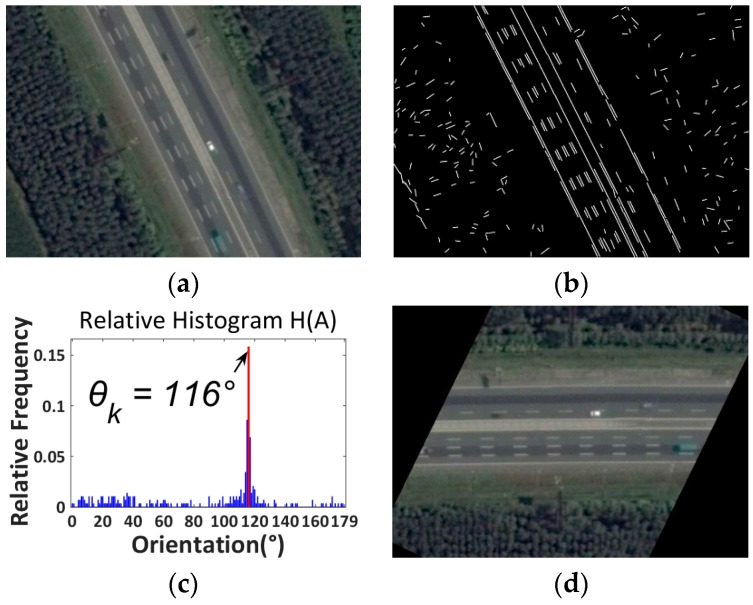
Low radiometric quality satellite image. (**a**) Color image; (**b**) Line segments detection using LSD; (**c**) Relative histogram (blue bins: distribution frequencies of relative histogram which correspond to different angles; red bin: the maximum distribution frequency of relative histogram which corresponds to the orientation of the road); (**d**) Rotated image.

The video images used for our testing have relatively high resolution of 1920 × 1080. But technically, our method can also work with images with low radiometric quality. Particularly, we performed our roadway orientation method on a low radiometric quality satellite image (see Figure 17). It can be seen from the figure that our method performed well on the image with low radiometric quality. Further comprehensive evaluation might be needed for future research.

### 5.4. Detection Using Oriented and Mosaicked Images

**Figure 18:**
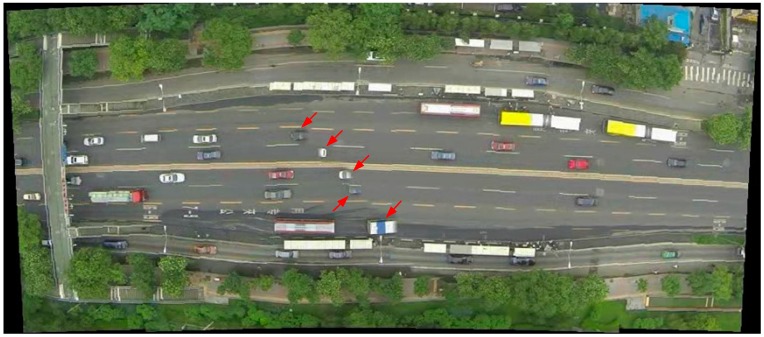
Mosaicked image. “Ghost” vehicles (marked by red arrows).

To be clear we did not use oriented, mosaicked images for detection in this research because mosaicked images may contain “ghost” vehicles [35], as marked by red arrows in Figure 18. For those “ghost” vehicles, only parts of the vehicle body can be seen. The reason for “ghost” vehicles is that the mosaicked image in Figure 18 was created by two different frames without synchronization (i.e., the two images were captured on different moments). Therefore those moving vehicles passing over the junction of the two images were cut off due to image mosaic. Obviously, those “ghost” vehicles will affect the accuracy of vehicle detections.

### 5.5. Vehicle Detection for Turning Vehicles

One drawback of the proposed vehicle detection method is its incapability of detecting turning vehicles. The roadway orientation adjustment can only rotate the image according to the orientation of the road. For turning vehicles, their orientations are changing during turning process and not aligned with the V-J and HOG + SVM detectors. This creates difficulties for vehicle detection. The original V-J and HOG + SVM methods also have this problem.

It is worth mentioning some recently developed state-of-the-art methods [19,20,21], which can detect vehicles in arbitrary directions. For example, Moranduzzo and Melgani [19] developed an automatic vehicle detection method, which is insensitive to vehicles’ in-plane rotation. Conceptually, this method is superior to our method, because this method can be used to detect turning vehicles, while our method cannot, but that method needs to extract asphalted areas (i.e., road regions) first, which could cause some difficulties in detection since in urban traffic environment with complicated road conditions (different road type, roadway surfaces, weather, and illumination), road regions might be difficult to detect without the need of additional resources, like GIS. Moranduzzo and Melgani [20] further improved their method by using an additional road database, but clearly the need of the support of additional road database might limit their applications. Overall, Moranduzzo and Melgani’s method achieved a total accuracy of 63.01%. When extracting road regions using GIS-based road maps, the total accuracy can be improved to 76.61%. Although this accuracy is lower than our method (88.50%), a comprehensive comparison using same images will be necessary. So far, due to lack of source codes of this method, such a comparison is difficult to perform. We will leave this work for future research.

Similarly, Liu and Mattyus [21] developed a multiclass vehicle detection method, which can detect vehicles with arbitrary orientations. To solve vehicle orientation problem, they trained a single classifier based on integral channel features (ICF) [36] which is able to detect vehicles orientated in different directions. Then eight binary classifiers are trained, each corresponding to a specific vehicle orientation. This method achieved a recall rate of 79.0% and a precision rate of 94%. Clearly, this method is competitive to our method with 88.5% recall rate and 92.15% precision rate. However, in Liu and Mattyus’ method, training the single classifier needs vehicle samples with different directions (i.e., eight different directions in [21]), therefore the sample library is very large. Furthermore, labeling the training samples requires a lot of experience and is a tedious and time-consuming task. In our opinion, for vehicle detections from UAV images captured over arterial roads, as the majority of vehicles run in the same direction with the road, it is reasonable to detect vehicles by a single detector which is sensitive to only one specific direction, as performed in our method. This could significantly reduce the work load on collecting training samples by nearly one order of magnitude, since only samples of one specific direction are needed. The precision rate of our method (92.15%) is a bit lower than that of [21] (94%); this could be the tradeoff between high precision rate and less work load. But we have to point out that Liu and Mattyus’ method can detect vehicles with arbitrary orientations, particularly for turning vehicles, while our method cannot. So conceptually Liu and Mattyus’ method is superior to our method. A comprehensive comparison using same images will be necessary; but so far, due to the lack of source codes of this method, such comparison is difficult to perform. We will leave this work for future research.

### 5.6. Sensitivity Analysis of Switching Interval T

**Figure 19:**
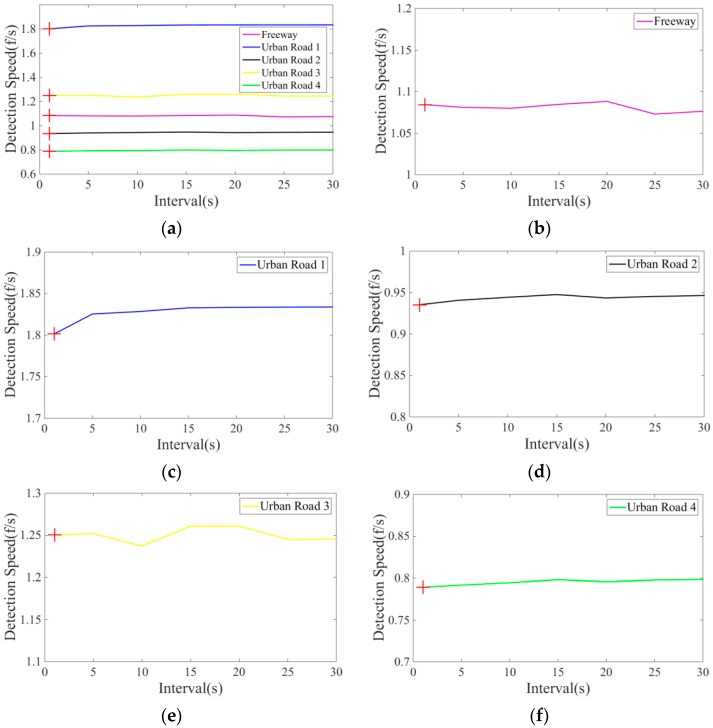
Vehicle detection speed of our method in different test scenes and the red cross shaped “+” presents the detection speed when the value of T is 24 (i.e., the interval is 1 s). (**a**) five scenes; (**b**) freeway; (**c**) urban road 1; (**d**) urban road 2; (**e**) urban road 3; (**f**) urban road 4.

During our testing, the switching time interval, T, was arbitrarily set as 24 (i.e., 1 s time interval) based on the assumption that traffic conditions will not change abruptly over a period of time. But it is very possible that different T values might lead to different detection speed and detection accuracy. To comprehensively analyze the influence of different intervals of T on the detection speed and accuracy, experiments with different *T* = {24,120,240,360,480,600,720} (namely, time intervals = 1, 5, 10, 15, 20, 25, 30 s) have been conducted. The testing was applied on the same datasets as shown in Table 1. The testing results of our method with different T intervals are shown in Table 3. It can be seen from Table 3 that, the influences of interval T on detection speed and accuracy are actually small. The reason would be that the time intervals for our videos are short and the traffic conditions do not change much, as confirmed in research done by [30]. Particularly, we present the speed changes of our method under different T intervals in Figure 19. Note the value of T we used for evaluating the performance of our method (see Table 2) is 24 (i.e., the interval is 1 s). For *T* = 24, the corresponding detection speeds were marked with red cross-shaped “+” shown in Figure 19 for comparison.

## 6. Concluding Remarks

In this paper, a new hybrid vehicle detection scheme which integrates V-J and HOG + SVM methods is proposed. As both V-J and HOG + SVM are sensitive to on-road vehicles’ in-plane rotation, the proposed scheme first adopts a roadway orientation adjustment method, which rotates each UAV image so that roads and on-road vehicles in images are aligned with the vehicle detector. After rotation, the original V-J or HOG + SVM methods can be applied to achieve higher accuracy. To address the issue of descending detection speed for both V-J and HOG + SVM, the proposed scheme further develops a hybrid and adaptive switching strategy which sophistically integrates V-J and HOG + SVM methods based on their different descending trends of detection speed to achieve better detection efficiency. A comprehensive evaluation shows that the switching strategy, combined with the road orientation adjustment method, can significantly improve the efficiency and effectiveness of the vehicle detection from UAV images. The results also show that the proposed vehicle detection method is competitive compared with other existing vehicle detection methods. Furthermore, it is worth mentioning that the proposed vehicle detection method can be performed on videos captured from moving UAV platforms without the need of image registration or additional road database, therefore it has great potentials of wide field applications.

However, the proposed vehicle detection scheme has difficulties to address turning vehicles. The future research will aim to address this problem. Some recently developed state-of-the-art methods [19,20,21,37] will be useful references for our future research. Particularly, the hybrid deep convolutional neural networks (DNNs) suggested by Chen et al. [37] would be an interesting direction for our future research. Indeed, it would be very interesting to compare our method with the DNN method, and to seek any possibility of applying the Faster R-CNN [38,39] for multimodal object detection (car, bus, truck, van, pedestrian, etc.) from UAV images. Due to the difficulty of constructing DNN and Faster R-CNN, we will leave all these for our future research.

## Figures and Tables

**Table 1:** Description of UAV Video Datasets.

Scenarios	Traffic Condition; Weather; Location; Time; Flight Altitude
Freeway	Non-congested; cloudy; G6 Jingzang Expressway; 15:40–16:40, 18 November 2014; 150 m.
Urban road 1	Non-congested; foggy; Xueyuan Road; 15:30–16:30, 26 March 2014; 170 m.
Urban road 2	Congested; cloudy; North Fourth Ring Road; 16:20–17:20, 6 November 2014; 150 m.
Urban road 3	Non Congested; cloudy; Xueyuan Road; 15:10–16:10, 12 August 2014; 100 m.
Urban road 4	Congested ; cloudy; North Fourth Ring Road; 16:30–17:30, 5 May 2015; 115 m.

**Table 2:** Vehicle Detection Results.

Scene	Metrics	ViBe	Frame Diff	V-J	V-J + 9	V-J + R	SVM	SVM + 9	SVM + R	V-J + SVM + S	V-J + SVM + R + S
Freeway	Cor. (%)	65.00%	61.11%	85.71%	87.50%	88.57%	91.30%	88.24%	92.27%	89.01%	89.37%
	Com. (%)	86.67%	78.57%	75.00%	92.11%	93.94%	55.26%	85.71%	87.04%	66.76%	94.24%
	Qua. (%)	59.09%	52.38%	66.67%	81.40%	83.78%	52.50%	76.92%	81.13%	61.67%	84.74%
	Speed (f/s)	5.59	9.96	1.16	0.064	0.96	1.17	0.083	1.03	1.17	1.08
Urban road 1	Cor. (%)	49.06%	77.78%	89.47%	73.53%	88.00%	82.05%	80.81%	85.64%	88.86%	87.89%
	Com. (%)	66.67%	48.84%	70.83%	92.59%	91.67%	69.57%	87.43%	90.96%	70.73%	91.46%
	Qua. (%)	39.39%	42.86%	65.38%	69.44%	81.48%	60.38%	72.40%	78.92%	62.89%	81.23%
	Speed (f/s)	6.64	10.40	2.08	0.16	1.57	1.15	0.079	1.03	2.01	1.80
Urban road 2	Cor. (%)	77.87%	75.26%	85.06%	92.16%	95.65%	98.67%	96.34%	98.01%	98.04%	96.39%
	Com. (%)	86.36%	68.87%	89.16%	94.95%	94.62%	77.08%	91.20%	92.96%	77.74%	94.24%
	Qua. (%)	69.34%	56.15%	77.08%	87.85%	90.72%	76.29%	88.14%	91.25%	76.55%	91.02%
	Speed (f/s)	6.17	10.01	0.50	0.049	0.43	1.02	0.065	0.87	1.00	0.94
Urban road 3	Cor. (%)	51.11%	53.49%	64.29%	40.54%	77.78%	66.39%	61.45%	75.94%	64.43%	78.77%
	Com. (%)	79.31%	85.19%	69.23%	88.24%	92.72%	84.21%	89.94%	90.70%	71.01%	92.16%
	Qua. (%)	45.10%	48.94%	50.00%	38.46%	73.30%	59.04%	57.50%	70.46%	51.01%	73.82%
	Speed (f/s)	6.22	10.26	1.37	0.063	0.97	1.16	0.077	1.04	1.36	1.25
Urban road 4	Cor. (%)	67.89%	77.53%	91.59%	86.76%	90.32%	90.99%	86.99%	90.92%	91.05%	90.09%
	Com. (%)	80.43%	51.49%	80.33%	93.65%	88.89%	78.91%	91.73%	93.96%	78.98%	88.64%
	Qua. (%)	58.27%	44.81%	74.81%	81.94%	81.16%	73.19%	80.67%	85.90%	73.29%	80.76%
	Speed (f/s)	5.27	9.64	0.60	0.056	0.46	0.85	0.066	0.75	0.83	0.79
Average	Cor. (%)	62.19%	69.03%	83.22%	76.10%	88.06%	85.88%	82.76%	88.56%	86.28%	88.50%
	Com. (%)	79.89%	66.59%	76.91%	92.31%	92.37%	73.01%	89.21%	91.13%	73.04%	92.15%
	Qua. (%)	54.24%	49.03%	66.79%	71.82%	82.09%	64.28%	75.13%	81.53%	65.08%	82.32%
	Speed (f/s)	5.98	10.05	1.14	0.079	0.88	1.07	0.074	0.942	1.27	1.17

**Table 3:** Vehicle Detection Results with Different Intervals.

Scene	Metrics	*T* = 24 (1 s)	*T* = 120 (5 s)	*T* = 240 (10 s)	*T* = 360 (15 s)	*T* = 480 (20 s)	*T* = 600 (25 s)	*T* = 720 (30 s)
Freeway	Cor. (%)	89.37%	88.76%	89.60%	88.96%	88.72%	89.66%	90.52%
	Com. (%)	94.24%	94.34%	93.37%	94.16%	93.72%	93.04%	91.57%
	Qua. (%)	84.74%	84.27%	84.24%	84.30%	83.74%	84.03%	83.55%
	Speed (f/s)	1.0843	1.0811	1.0800	1.0847	1.0882	1.0730	1.0763
Urban road 1	Cor. (%)	87.89%	87.21%	88.00%	87.84%	87.17%	87.50%	87.21%
	Com. (%)	91.46%	91.65%	91.67%	91.23%	91.07%	91.42%	91.09%
	Qua. (%)	81.23%	80.79%	81.48%	81.00%	80.30%	80.86%	80.36%
	Speed (f/s)	1.8014	1.8255	1.8285	1.8329	1.8334	1.8337	1.8339
Urban road 2	Cor. (%)	96.39%	96.31%	96.90%	97.22%	96.59%	96.51%	96.36%
	Com. (%)	94.24%	94.13%	93.98%	93.58%	93.10%	92.28%	92.87%
	Qua. (%)	91.02%	90.86%	91.24%	91.15%	90.14%	89.30%	89.72%
	Speed (f/s)	0.9352	0.9406	0.9442	0.9475	0.9434	0.9453	0.9463
Urban road 3	Cor. (%)	78.77%	78.33%	78.75%	78.49%	77.65%	77.72%	77.47%
	Com. (%)	92.16%	91.26%	92.36%	91.53%	91.45%	91.78%	91.56%
	Qua. (%)	73.82%	72.87%	73.94%	73.18%	72.40%	72.66%	72.31%
	Speed (f/s)	1.2505	1.2519	1.2372	1.2608	1.2610	1.2453	1.2454
Urban road 4	Cor. (%)	90.09%	90.41%	89.93%	90.26%	90.30%	90.33%	90.52%
	Com. (%)	88.64%	89.42%	90.19%	91.13%	91.17%	91.80%	92.70%
	Qua. (%)	80.76%	81.68%	81.91%	82.97%	83.04%	83.58%	84.49%
	Speed (f/s)	0.7893	0.7918	0.7946	0.7981	0.7957	0.7977	0.7984
Average	Cor. (%)	88.50%	88.20%	88.64%	88.55%	88.09%	88.34%	88.41%
	Com. (%)	92.15%	92.16%	92.31%	92.32%	92.10%	92.06%	91.96%
	Qua. (%)	82.32%	82.09%	82.56%	82.52%	81.92%	82.08%	82.09%
	Speed (f/s)	1.1722	1.1782	1.1769	1.1848	1.1843	1.1790	1.1801

## Supplementary Materials

The supplementary materials are available online at http://www.mdpi.com/1424-8220/16/8/1325/s1.

## Abbreviations

UAV	unmanned aerial vehicle
SVM	support vector machine
HOG	histogram of oriented gradient
LSD	line segment detector
DPM	deformable part model
V-J	Viola-Jones object detection scheme
HOG + SVM	linear SVM classifier with HOG feature
Cor.	*Correctness*
Com.	*Completeness*
Qua.	*Quality*

## References

[1] Rosser K., Pavey K., Fitzgerald N., Fatiaki A., Neumann D., Carr D., Hanlon B., Chahl J. (2015). Autonomous Chemical Vapour Detection by Micro UAV. Remote Sens..

[2] Gonzalez L.F., Montes G.A., Puig E., Johnson S., Mengersen K., Gaston K.J. (2016). Unmanned Aerial Vehicles (UAVs) and Artificial Intelligence Revolutionizing Wildlife Monitoring and Conservation. Sensors.

[3] Boccardo P., Chiabrando F., Dutto F., Tonolo F.G., Lingua A. (2015). UAV Deployment Exercise for Mapping Purposes: Evaluation of Emergency Response Applications. Sensors.

[4] Agrawal A., Hickman M. Automated extraction of queue lengths from airborne imagery. Proceedings of the International IEEE Conference on Intelligent Transportation Systems.

[5] Coifman B., Mccord M., Mishalani R.G., Iswalt M. (2006). Roadway traffic monitoring from an unmanned aerial vehicle. IEE Proc. Intell. Transp. Syst..

[6] Yu G., Zhou B., Wang Y., Wu X., Wang P. (2015). Measuring algorithm for the distance to a preceding vehicle on curve road using on-board monocular camera. Int. J. Bifurc. Chaos.

[7] Angel A., Hickman M., Mirchandani P., Chandnani D. (2003). Methods of analyzing traffic imagery collected from aerial platforms. IEEE Trans. Intell. Transp. Syst..

[8] Azevedo C.L., Cardoso J.L., Ben-Akiva M., Costeira J.P., Marques M. (2014). Automatic Vehicle Trajectory Extraction by Aerial Remote Sensing. Procedia Soc. Behav. Sci..

[9] Shastry A.C., Schowengerdt R.A. (2005). Airborne video registration and traffic-flow parameter estimation. IEEE Trans. Intell. Transp. Syst..

[10] Yalcin H., Hebert M., Collins R., Black M.J. A Flow-Based Approach to Vehicle Detection and Background Mosaicking in Airborne Video. Proceedings of the 2005 IEEE Computer Society Conference on Computer Vision and Pattern Recognition.

[11] Viola P., Jones M. Rapid object detection using a boosted cascade of simple features. Proceedings of the 2001 IEEE Computer Society Conference on Computer Vision and Pattern Recognition.

[12] Dalal N., Triggs B. Histograms of oriented gradients for human detection. Proceedings of the 2005 IEEE Computer Society Conference on Computer Vision and Pattern Recognition.

[13] Cao X., Wu C., Yan P., Li X. Linear SVM classification using boosting HOG features for vehicle detection in low-altitude airborne videos. Proceedings of the IEEE International Conference on Image Processing.

[14] Cao X., Wu C., Lan J., Yan P. (2011). Vehicle Detection and Motion Analysis in Low-Altitude Airborne Video Under Urban Environment. IEEE Trans. Circuits Syst. Video Technol..

[15] Leitloff J., Rosenbaum D., Kurz F., Meynberg O., Reinartz P. (2014). An Operational System for Estimating Road Traffic Information from Aerial Images. Remote Sens..

[16] Tuermer S., Kurz F., Reinartz P., Stilla U. (2013). Airborne Vehicle Detection in Dense Urban Areas Using HoG Features and Disparity Maps. IEEE J. Sel. Top. Appl. Earth Obs. Remote Sens..

[17] Xu Y., Yu G., Wang Y., Wu X. Vehicle Detection and Tracking from Airborne Images. Proceedings of the 15th COTA International Conference of Transportation Professionals.

[18] Jones M., Viola P. Fast Multi-view Face Detection. Proceedings of the 2003 IEEE Computer Society Conference on Computer Vision and Pattern Recognition.

[19] Moranduzzo T., Melgani F. (2014). Detecting cars in UAV images with a catalog-based approach. IEEE Trans. Geosci. Remote Sens..

[20] Moranduzzo T., Melgani F. (2014). Automatic car counting method for unmanned aerial vehicle images. IEEE Trans. Geosci. Remote Sens..

[21] Liu K., Mattyus G. (2015). Fast Multiclass Vehicle Detection on Aerial Images. IEEE Geosci. Remote Sens. Lett..

[22] Felzenszwalb P.F., Girshick R.B., McAllester D., Ramanan D. (2010). Object Detection with Discriminatively Trained Part Based Models. IEEE Trans. Pattern Anal. Mach. Intell..

[23] Friedman J., Hastie T., Tibshirani R. (2000). Additive Logistic Regression: A Statistical View of Boosting. Ann. Stat..

[24] Cucchiara R., Piccardi M., Mello P. (2000). Image analysis and rule-based reasoning for a traffic monitoring system. IEEE Trans. Intell. Transp. Syst..

[25] Tao H., Sawhney H., Kumar R. (2002). Object tracking with Bayesian estimation of dynamic layer representations. IEEE Trans. Pattern Anal. Mach. Intell..

[26] Ma Y., Wu X., Yu G., Xu Y., Wang Y. (2016). Pedestrian Detection and Tracking from Low-Resolution Unmanned Aerial Vehicle Thermal Imagery. Sensors.

[27] Grompone V.G.R., Jakubowicz J., Morel J.M., Randall G. (2010). LSD: A Fast Line Segment Detector with a False Detection Control. IEEE Trans. Pattern Anal. Mach. Intell..

[28] Ballard D.H. (1981). Generalizing the Hough Transform to Detect Arbitrary Shapes. Pattern Recognit..

[29] Gioi R.G.V., Jakubowicz J., Morel J.M., Randall G. (2012). LSD: A Line Segment Detector. Image Process. Line.

[30] Maerivoet S., De Moor B. (2005). Traffic Flow Theory. Physics.

[31] Olivier B., Marc V.D. (2011). ViBe: A universal background subtraction algorithm for video sequences. IEEE Trans. Image Process..

[32] Faugeras O., Deriche R., Mathieu H., Ayache N.J., Randall G. (1992). The Depth and Motion Analysis Machine. Int. J. Pattern Recognit. Artif. Intell..

[33] Burns J.B., Hanson A.R., Riseman E.M. (1986). Extracting Straight Lines. IEEE Trans. Pattern Anal. Mach. Intell..

[34] Canny J. (1998). A computational approach to edge detection. IEEE Trans. Pattern Anal. Mach. Intell..

[35] Uyttendaele M., Eden A., Skeliski R. Eliminating Ghosting and Exposure Artifacts in Image Mosaics. Proceedings of the 2001 IEEE Computer Society Conference on Computer Vision and Pattern Recognition.

[36] Dollár P., Tu Z., Perona P., Belongie S. Integral Channel Features. Proceedings of the British Machine Vision Conference.

[37] Chen X., Xiang S., Liu C.L., Pan C.H. (2014). Vehicle detection in satellite images by hybrid deep convolutional neural networks. IEEE Geosci. Remote Sens. Lett..

[38] Ren S., He K., Girshick R., Sun J. Faster R-CNN: Towards Real-Time Object Detection with Region Proposal Networks. Proceedings of the Neural Information Processing Systems Conference.

[39] Faster R-CNN. https://github.com/rbgirshick/py-faster-rcnn.

