# Supplementary figures and images for: A Hybrid Vehicle Detection Method Based on Viola-Jones and HOG + SVM from UAV Images
Source: Sensors (Basel). 2016 Aug 19;16(8):1325. doi: 10.3390/s16081325 (PMC5017490; doi:10.3390/s16081325)

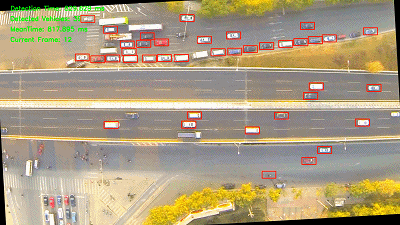

Supplement: Supplementary file 1 [file sensors-16-01325-s001.zip › supplementary/Vehicle Detection-1.gif]

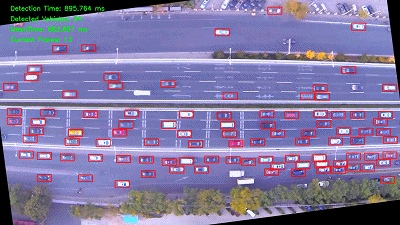

Supplement: Supplementary file 1 [file sensors-16-01325-s001.zip › supplementary/Vehicle Detection-2.gif]
